# The effect of exercise on aromatase inhibitor-induced musculoskeletal symptoms in breast cancer survivors :a systematic review and meta-analysis

**DOI:** 10.1007/s00520-019-05186-1

**Published:** 2019-12-18

**Authors:** Geling Lu, Jin Zheng, Lei Zhang

**Affiliations:** 1 Department of Urology, The First Hospital of China Medical University, Liaoning, China; 2 Department of Breast, The First Hospital of China Medical University, Liaoning, China

**Keywords:** Breast cancer, Exercise, Aromatase inhibitor, Musculoskeletal symptoms, Meta-analysis

## Abstract

**Background:**

Evidence is mixed regarding the effect of exercise programs on improving musculoskeletal symptoms and quality of life. Previous meta-analyses have not focused specifically on the musculoskeletal symptoms. Therefore, this meta-analysis aimed to evaluate the effect of exercise on these outcomes in breast cancer survivors taking aromatase inhibitors.

**Methods:**

PubMed, CINAHL, EMBASE, Web of Science, Wan Fang, CNKI, VIP, and CBM were searched for randomized control trials or quasi-experimental studies from the establishment of the database to May 2019. Studies comparing exercise programs with usual care among breast cancer survivors taking aromatase inhibitors were included. The primary outcome was the degree of musculoskeletal symptoms, as assessed by scores of pain, stiffness, and grip strength. The secondary outcome was the total quality of life score.

**Results:**

A total of 9 studies involving 743 participants were included. Exercise programs were more effective than usual care in improving musculoskeletal symptoms among breast cancer patients taking AIs. The subgroup scores of pain (SMD = -0.46, 95% CI -0.79 to -0.13, P = 0.006), stiffness (SMD = -0.40, 95% CI -0.71 to -0.08, P = 0.01), and grip strength (SMD = 0.43, 95% CI 0.16 to 0.71, P = 0.002) benefited from exercise interventions. Similar effects were found for the quality of life scores (SMD = 2.24, 95% CI 0.28 to 4.21, P = 0.03).

**Conclusions:**

Results indicate that exercise relieves musculoskeletal symptoms and improves quality of life, which can be used to motivate patients to exercise actively under professional guidance. Due to a small sample size, further research is required to ensure the effectiveness of exercise on musculoskeletal symptoms and quality of life.

## Introduction

Breast cancer is the most prevalent cancer in women worldwide [1]. Approximately 75% of breast cancer survivors are diagnosed with hormone-receptor-positive breast cancer [2]. As a part of standard adjuvant therapy for postmenopausal women diagnosed with hormone-receptor-positive breast cancer [3], aromatase inhibitors (AIs) have improved disease-free survival by 40%; however, they also cause aromatase inhibitor-associated musculoskeletal symptoms (AIMSS) [4]. AIMSS occur in up to 54% of patients, presenting as mild or moderate joint pain, muscle stiffness, decreased grip strength, and so on [5]. AIMSS are the most common reason for postmenopausal breast cancer survivors to discontinue AI treatment [6, 7]. Self-discontinuation of AIs because of side effects may attenuate the efficacy of AIs, thereby increasing the likelihood of cancer recurrence [8].

Currently, the management of AIMSS can be categorized into pharmacological and nonpharmacological therapies. Although duloxetine has a greater reduction in AIMSS, it leads to frequent adverse events, such as dry mouth, nausea, headache, and fatigue [9]. There is a lack of clinical trials that prove the safety of pharmacological therapies, so drugs cannot be widely used in clinical practice for a long period of time [10]. Yoga significantly reduced muscle aches, general pain, and physical discomfort among breast cancer survivors on hormone therapy [11]. As one of the nonpharmacological treatments, exercise programs have received much attention in recent years. An exercise program [12] refers to the prevention and improvement of AIMSS through aerobic exercise, resistance exercise, or a combination of both, and it includes activities such as yoga, walking, and swimming. Exercise intensity should be guided by an exercise trainer at a safe and comfortable pace or no more than 80% of heart rate reserve. However, the effect of exercise on AIMSS remains inconclusive. Many original studies have noted the effectiveness of exercise on AIMSS and quality of life (QOL) among breast cancer survivors taking AIs [13–15], while other researches have not reached the same conclusion [12, 16–19]. It is necessary to perform a systematic review and meta-analysis on the effect of exercise on AIMSS and QOL.

Previous systematic reviews about the effect of exercise among breast cancer survivors were conducted with searches in PubMed, the Joanna Briggs Institute (JBI), the Cochrane Library, and other electronic databases. Exercise programs could significantly improve subjective sleep problems, decrease fatigue, and control sex hormones [20–22], but none of the meta-analyses directly examined the effect of exercise on AIMSS. Thus, the current study aimed to conduct a meta-analysis on the effect of exercise on AIMSS and QOL in breast cancer survivors with AIs.

## Methods

### Design

This systematic review was conducted according to the guidelines of the Cochrane Collaboration [23], and the report was based on the principles of the PRISMA statement [24].

### Data sources and searches

The following electronic databases were searched from their inception to May 1^st^, 2019: PubMed, Web of Science, EMBASE, CINAHL, Chinese Biomedical Service Platform (CBM), Wan Fang, Chinese Scientific and Technological Journal Database (VIP), and China National Knowledge Infrastructure (CNKI). Searches were restricted to studies in English and Chinese.

The main search terms used to search the databases included “breast cancer/breast neoplasms/breast tumor/breast carcinoma, exemestane/letrozole/aminoglutethimide/anastrozole/formestane/aromatase inhibitors/aromatase inhibitor, exercise/physical activity/aerobic exercise/yoga/tai chi/walking/jogging/dance, musculoskeletal/musculoskeletal/ pain/stiffness” and so on. Free terms and keywords from the thesaurus of each database were combined. Two authors screened the studies, and they resolved disagreements via discussion or by referring to another author if necessary.

The retrieval strategy consisted of the following four steps. First, a related systematic review and meta-analysis from JBI and the Cochrane Library were conducted. Second, related original research was searched in the databases, reading the title, and abstract after removing duplicate articles by EndnoteX8 software. Third, the full text of articles that initially met the inclusion criteria was read. Last, the references of the included research studies were searched.

### Study selection

Studies were eligible for inclusion if they were randomized control trials or quasi-experimental studies. Included studies examined the effectiveness of any types of exercise on AIMSS or QOL in breast cancer patients taking AIs. Two authors independently screened the studies, and they resolved disagreements via discussion or referring to the third author if necessary.

### Participants

Participants had a diagnosis of breast neoplasms in accordance with diagnostic criteria [25] and were taking AIs. The severity of AIMSS was not limited. Individuals with other malignant tumors or metastatic breast cancer, and arthritis or joint pain attributed to inflammatory arthritis (such as rheumatoid arthritis, osteoarthritis, or gout) prior to hormone therapy were excluded. Those unable to exercise due to mobility issues were excluded.

### Intervention

The experimental group had to exercise as defined previously. Exercise programs required specialists’guidance, although the duration, frequency, and intensity, as well as whether they were home-based or group exercise programs, were not restricted.

### Comparison

Individuals in the control group were told to continue their usual activities, or they received convention care. They were not given exercise instruction until the end of the study.

### Outcomes

The included studies had to measure at least one of the outcomes: subgroup scores for AIMSS or the total QOL score. Subgroup scores of AIMSS consisted of three types of symptoms: pain, stiffness, and grip strength. All the outcomes were measured with continuous data. The score of AIMSS was measured using the BPI scale, WOMAC scale, VAS scale, electronic algometer, and so on. The QOL score was measured by self-reported measurements, including the FACT scale and SF36 scale.

### Quality assessment

The quality assessment was conducted by two authors independently. The Cochrane Handbook for Systematic Reviews of Interventions Version 5.3.0 of the Cochrane Collaboration [26] was used to assess the risk of bias in the included studies and included random sequence generation (selection bias), allocation concealment (selection bias), blinding of participants and personnel (performance bias), blinding of outcome assessment (detection bias), incomplete outcome data (attrition bias), selective reporting (reporting bias), and other bias. The results of the assessment were classified as low risk, unclear risk, or high risk. The authors resolved disagreements by discussion and, if necessary, by referring to another author.

### Data extraction

After reading the full text of the eligible studies, the following characteristics and outcome data were extracted by two authors: author, publication year, country/area where the study was performed, study design, sample size and mean age range, intervention of experimental and control groups, intervention details, follow-up, and the mean and standard deviation of outcomes (the score of AIMSS, including pain, stiffness, and grip strength, and the QOL score). If standard deviations were not reported in the original studies, the confidence interval (CI) and *P* value provided were converted into standard deviation according to the method described in the Cochrane Handbook.

### Statistical analysis

Data were pooled for statistical meta-analysis via RevMan 5.3 provided by the Cochrane Collaboration. All the outcomes were continuous data and expressed as a 95% confidence interval (CI) with a significance level of α = 0.05. The mean difference (MD) was calculated as an effect measure when the pooled studies used the same scale, and the standardized mean difference (SMD) was calculated when the pooled studies used different rating scales to assess outcomes. Statistical heterogeneity was tested by chi-square and I^2^. Random-effects models were used when *P* < 0.10 and I^2^ > 50%. Fixed-effects models were used when *P* > 0.10 and I^2^ < 50%. If there was a significant clinical heterogeneity between studies, descriptive analysis was performed. Sensitivity analysis was used to test the stability of the results.

## Results

### Literature search

**Fig. 1:**
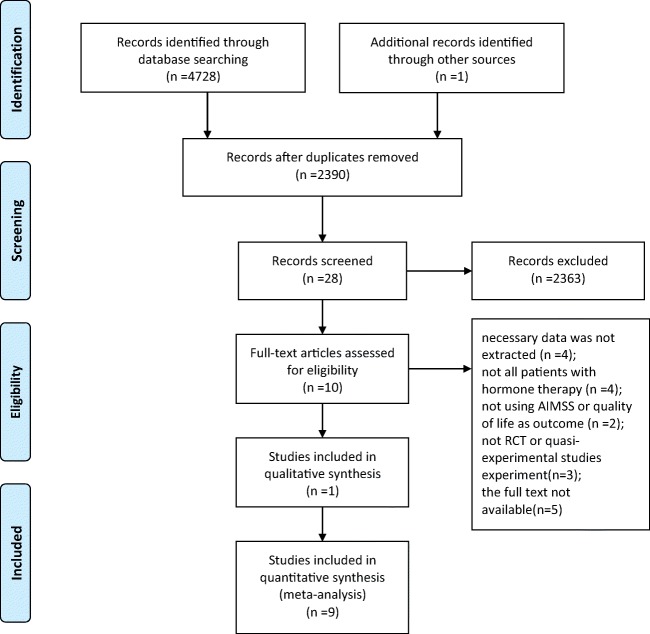
Flow chart diagram of trial identification and selection

There were nine studies included in the meta-analysis. A total of 4728 studies were retrieved from databases, and 2390 studies remained after removing the duplications via EndnoteX8 software. After reading the title and abstract, 2263 studies were excluded. After reviewing the full text of the remaining 27 articles, 8 publications were eligible for inclusion in this study [14, 27–32, 34]. One further study [33] was found through the references of the eligible publications. The retrieval process is shown in Fig. 1.

### Characterization of the included studies

In the present analysis, 9 studies (8 RCTs and 1 quasi-experimental study) with a total of 743 participants were included. The studies were performed in the USA [27–29, 33], Spain [14], England [34], and China [30, 31]. The number of participants ranged from 40 to 121 and the mean age of the participants varied between 32 and 72 years old. The types of interventions included walking, aquatic exercise, strength training, bench press, leg press, seated row, and so on.

Aerobic exercise was performed in all studies, five of which included resistance exercise [14, 27, 28, 31, 32]. The duration of the interventions ranged from six weeks to 12 months, with at least 120 mins/week of exercise prescribed. Training intensity varied among studies ranging from 60 to 80% of predicted maximal heart rate. There were no major adverse effects reported in the included studies. Nikander et al. [33] reported joint and muscle pain as well as stiffness because of overuse. All these symptoms were relieved after 1-2 weeks, and participants finished the exercise program. Further details about the included studies are shown in Table 1.

**Table 1:** Characteristics of the included studies

Study	Country	Design	Description ofbreastcancer/AIMSS	Participants	Intervention	Intervention details	Comparison	Follow-up	Outcomes
Baglia et al. [27] 2019	USA	RCT	breast cancer(stage 1-3) /atleast mildarthralgia	N = 121exp = 61 age = 62.0±7.0con = 60 age = 60.5±7.0	combinedexercise/supervised,group-based and home-based (walking)	length = 12 m Duration = 150 min/weekFrequency = 3 or 5 sessions/week Intensity = noreport	continue usual activities andoffer exercise instructionafter the end of the study	baseline、6 m、12 m	SF36、FACT-ES、FACT-B、FACT-G、intervention adherence
Tan et al. [31] 2018	China	RCT	breast cancer(stage 1-3)/notreport	N = 74exp = 37 age = 59.5±1.4con = 37 age = 59.4±1.5	combinedexercise/supervised(progressive walking)	length = 15 w Duration = at least 170 min/weekFrequency = 2 times/week Intensity = 45-65%	not offer instruction	baseline、15 w	VAS、comprehensive assessmentquestionnaire、the level of blood lipidand blood pressure 、DASH scale
Nyrop et al. [29] 2017	USA	RCT	breast cancer(stage 1-4)/moderateAIMSS	N = 62 age = 63.8±8.3exp = 31 age = 63.3±6.9con = 31 age = 64.4±9.7	aerobic exercise/ home-based or group-based(walking program)	length = 6w Duration = 150 min/weekFrequency = daily Intensity = at a safe andcomfortable pace	continue usual activities andoffer exercise instructionafter the end of the study	baseline、6 w、6 m	VAS、WOMAC、FACT-G、RAI、OEE
Thomas etal. [28] 2017	USA	RCT	breast cancer(stage 1-3)/atleast mildarthralgia	N = 121exp = 61 age = 62.0±7.0con = 60 age = 60.5±7.0	combinedexercise/supervised, home-based (brisk walking)	length = 12 m Duration = 150 min/weekFrequency = twice-weekly Intensity = 60%-80%ofpredicted maximal heart rate	continue with their usualactivities	baseline、6 m、12 m	BPI、body composition
Fields et al. [34] 2016	UK	RCT	breast cancerwith pain	N = 40 age = 63±8exp = 20 age = 60±8con = 20 age = 66±7	aerobic exercise/supervisedgroup (Nordic walkingprogram)	length = 12w Duration = 120 min/weekFrequency = 4 sessions/week Intensity = at a safe andcomfortable pace	usual care and offer thechance to participate in aNordic walking program	baseline、6 w、12 w	BPI、SF36、PSEQ
Irwin et al. [32] 2015	USA	RCT	breast cancer(stage 1-3)/recerving anAI at least 6months andreport joint pain	N = 121exp = 61 age = 62.0±7.0con = 60 age = 60.5±7.0	combinedexercise/superised (benchpress, latissimus pull down,seated row, leg press, legextension, leg curl)	length = 12 m Duration = 150 min/weekFrequency = twice/week Intensity = 60-80% of heart ratereserve	encourage to continue theirusual activities	baseline、6 m、12 m	BPI、WOMAC、grip strength、intervention adherence、AI adherence
Cantarero-Villanueva etal. [14] 2013	Spain	RCT	breast cancer(stage 1-3a)/reportarthralgia	N = 40exp = 20 age = 48.4±10.8con = 20 age = 46.2±7.4	combinedexercise/supervised group(aquatic exercise)	length = 2 m Duration = 3 days/weekFrequency = 8 sessions/week Intensity = no report	encourage to maintain theirusual activities	baseline、8 wbaseline、12 m	electronic algometer、Piper FatigueScale、body composition
Nikander et al. [33] 2012	USA	RCT	breast cancer(stage 1-3)/notreport	N = 86exp = 40 age = 53.7±6.8con = 37 age = 52.6±7.1	aerobicexercise/supervised/groupexerise and home training(dumbell exercise)	length = 12 m Duration = 150min/weekFrequency = 3 sessions/week Intensity = no report	encourage to maintain theirusual activities	baseline、15 w	isometric leg press、anthropometry、body composition、bone traits
Wu et al. [30] 2011	China	quasi-experimentalstudy	breast cancer/notreport	N = 78 age = 32-65 yexp = 39con = 39	aerobic exercise/home-based (body buildingexercise	length = 15 w Duration = 60-90 min/weekFrequency = 2-3 times/week Intensity = 50-70% of heartrate reserve	exercise was not restrictedfor 15 weeks and receivedthe same exerciseinstruction after 15 weeks	Follow-up	FLIC scale, range of motion

**Abbreviations:** BPI, Brief Pain Inventory; WOMAC, Western Ontario and McMaster University Osteoarthritis Index; DASH, Disabilities of the Arm, Shoulder and Hand; FACT-G, The FACT General; FACT-B, The FACT for patients with breast cancer; FACT-ES,FACT Endocrine Subscale; VAS, Visual Analog Scales; ASE, arthritis self-efficacy scale; RAI, Rheumatology Attitudes Index; SEPA, Self-efficacy for physical activity; OEE, outcome expectations; PESQ, Pain Self-efficacy Questionnaire; FLIC, the functional LivingIndex Cancer; SF-36, 36-Item Short Form Survey.

### Quality of methodology of the included studies

**Fig. 2:**
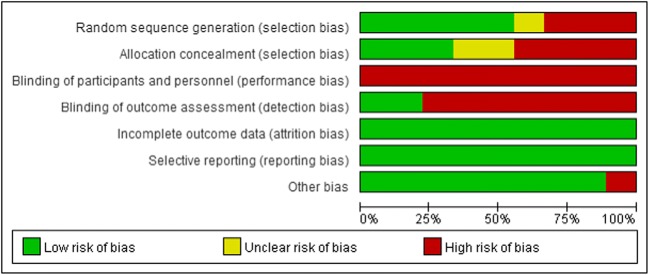
Overall risk of bias assessment using the Cochrane tool

**Fig. 3:**
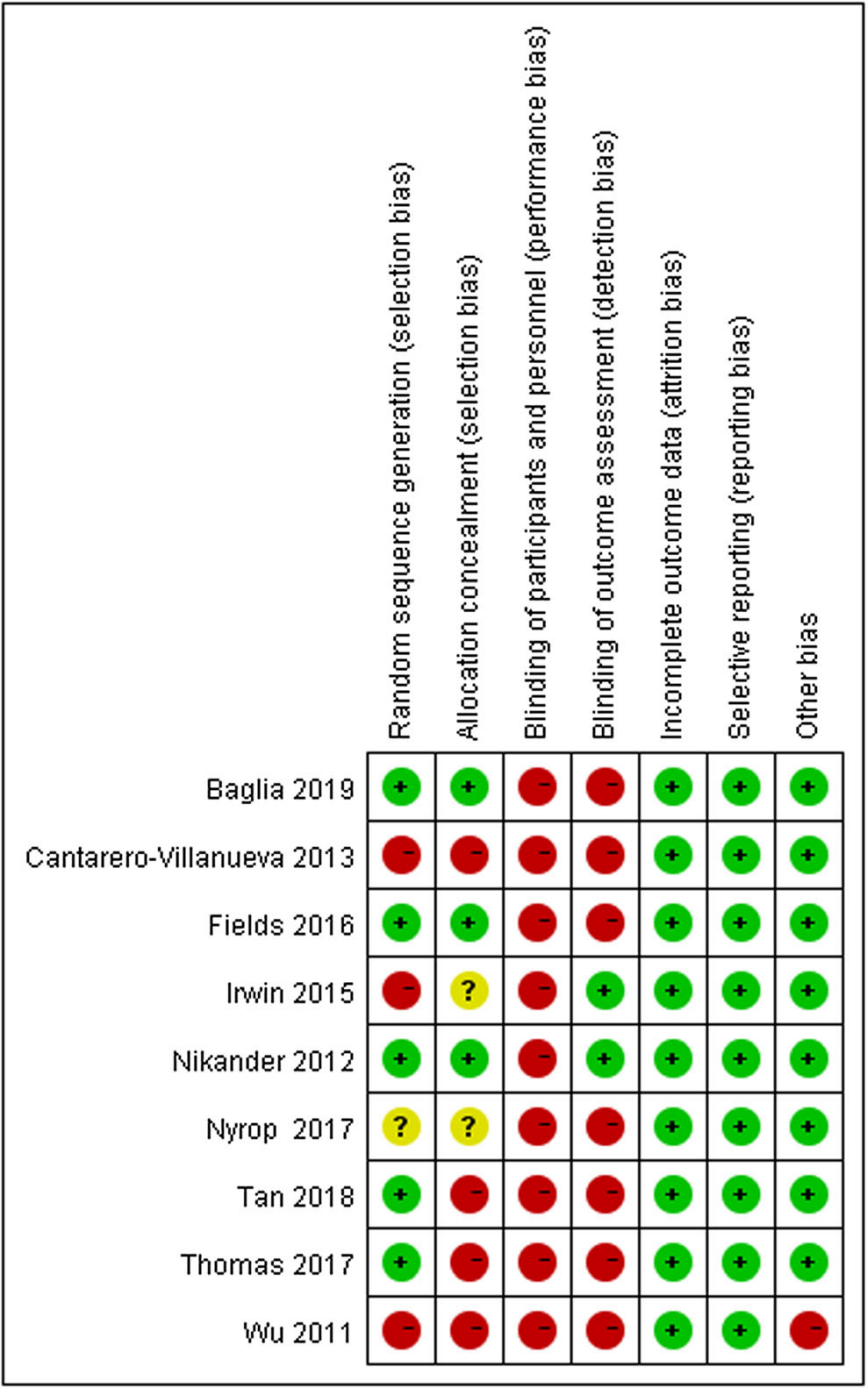
Risk of bias assessment by individual trials

Five studies provided specific information on how the random sequence was generated [27, 28, 31, 33, 34]. Three provided sufficient information on allocation concealment [27, 33, 34]. Because the nature of the intervention precluded blinding patients and personnel administering the intervention, all studies were considered to be at high risk of bias. All included studies reported the data on all outcomes measured, so the selective outcome reporting bias was regarded as low risk of bias. The results are shown in Fig. 2 and 3.

### Effects of exercise on the total and subgroup scores of AIMSS

Eight of the included studies measured the total score of AIMSS. In view of the different scales used in the study, SMD was used as the effect measure. The results showed that the effect of exercise on the management of AIMSS was significant.

**Fig. 4:**
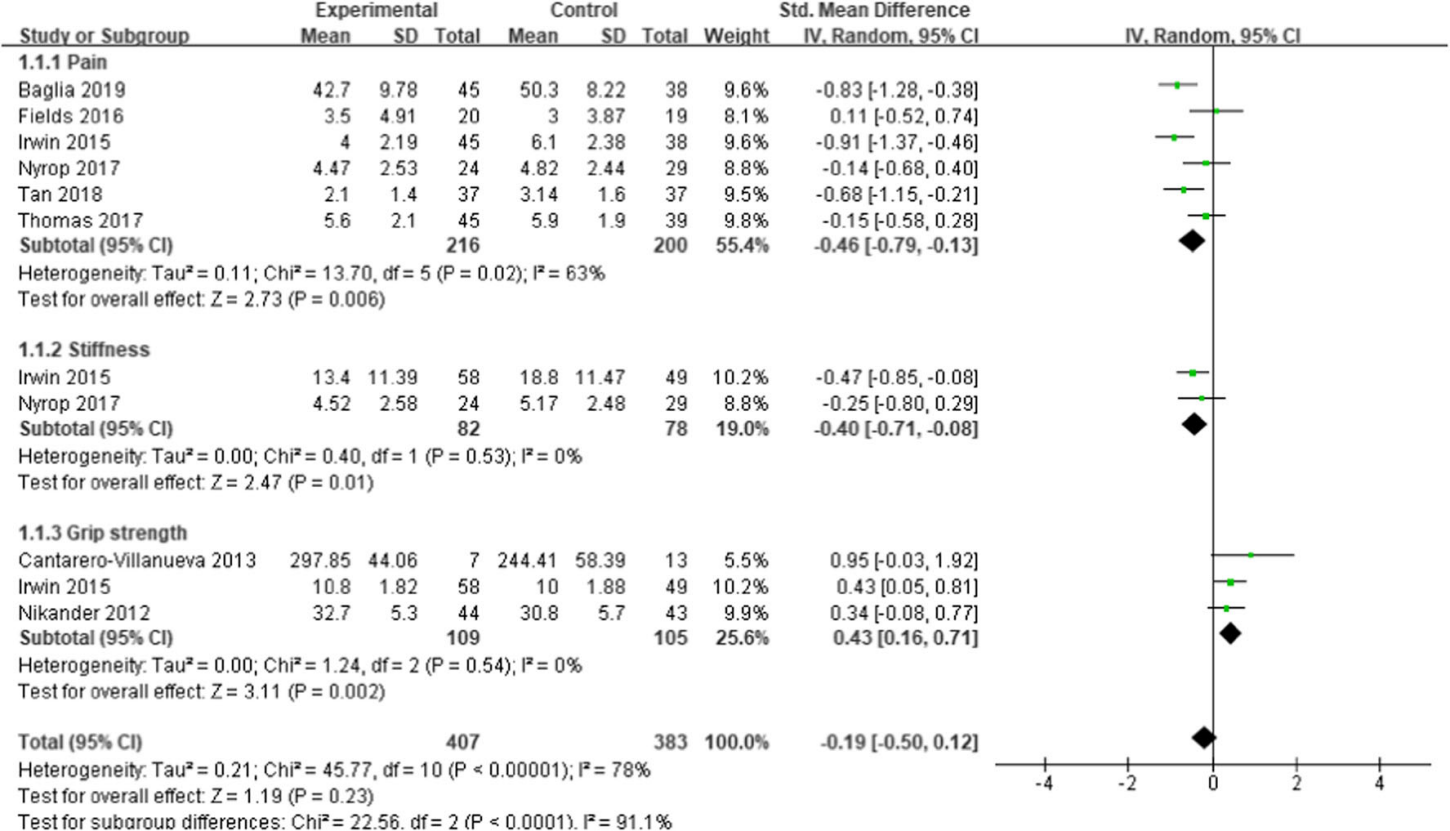
The effect of exercise on changes of AIMSS in breast cancer survivors

To reduce clinical heterogeneity, subgroup analysis was conducted for different symptoms: subgroup 1 was the comparison of pain, subgroup 2 was the comparison of stiffness, and subgroup 3 was the comparison of grip strength. Six studies [27–29, 31, 32, 34] evaluated the subgroup score for pain, two studies [29, 32] measured the subgroup score for stiffness, and three studies [14, 32, 33] measured the subgroup score for grip strength. The results revealed a statistically significant effect in favor of exercise on the management of pain [SMD = -0.46, 95% CI -0.79 to -0.13, *P* = 0.006], stiffness [SMD = -0.40, 95% CI -0.71 to -0.08, *P* = 0.01], and grip strength [SMD =0.43, 95% CI 0.16 to 0.17, *P* = 0.002], with statistical heterogeneity of 63, 0, and 0%, respectively. Only when the study of Irwin et al.[32]was omitted from the analysis in the subgroup of grip strength did the results would not show a significant effect [SMD = 0.48,95% CI (-0.02, 0.97), *P* = 0.06] (see Fig. 4 for further details).

### Effects of exercise on the total QOL score

**Fig. 5:**
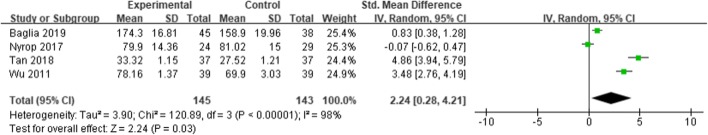
The effect of exercise on quality of life in breast cancer survivors

Four of the included studies [27, 29–31] measured the QOL score. The results showed that exercise had a favorable effect on QOL [SMD = 2.24, 95% CI 0.28 to 4.21, *P* = 0.03]. The results changed when we omitted the study of Tan et al. [31] [SMD = 1.12, 95% CI (-0.07, 2.32), *P* = 0.07], Baglia et al. [27] [SMD = 2.74, 95% CI (-0.30, 5.77), *P =* 0.08], and Wu et al. [30] [SMD = 1.83, 95% CI (-0.39, 4.06), *P* = 0.11] in the QOL analysis (see Fig. 5 for further details).

## Discussion

The current meta-analysis aimed to evaluate the effect of exercise on AIMSS and QOL. Regarding the blinding of participants, all studies were considered to be at high risk for performance bias because it is easy for participants to be aware of exercise. Blinding of the assessments was not possible because the primary outcome questionnaire was self-reported, except for grip strength. All studies compared baseline characteristics, such as age, education, and disease status, between the experimental group and the control group, and these characteristics were comparable. The outcomes were AIMSS and QOL, and the measurement tools varied.

The results of the meta-analysis indicate that exercise is a beneficial therapy that has a favorable effect on the management of AIMSS, in the areas of pain, stiffness, and grip strength, and on QOL in breast cancer patients taking AIs. The results were consistent with the results of previously published meta-analyses. Qiu et al. and Mishra et al. [35, 36] found that exercise was an effective intervention to improve quality of life in breast cancer survivors. Li et al. [37] concluded that strength training exercises could improve muscle strength. The current meta-analysis was comprehensive and included different interventions, both single exercises and combined exercises. Additionally, this study included participants who were postmenopausal women diagnosed with hormone-receptor-positive breast cancer, a group with a high incidence of AIMSS. To ensure patient safety, exercise was recommended under professional guidance. In the subgroup analysis, exercise was associated with a significant reduction in pain and stiffness and an improvement in grip strength. Similar results have been published. Strasser et al. [38] found that resistance exercise had positive effects on muscular function and body composition in cancer patients.

Various theoretical models have been proposed to explain the possible positive effects of exercise on breast cancer survivors taking aromatase inhibitors, although the variety of exercise types and different outcome measurement tools have lacked uniformity. Estrogen depletion has been suggested to be related to AIMSS [39]. Estrogen has attenuated pain via opioid neurons in the spinal cord; hence abruptly lowering the level of the estrogen would render patients taking AIs more sensitive to pain [40]. Exercise might play a role in increasingly releasing anti-inflammatory cytokines, while inflammation triggers hyperalgesia [41]. Kida et al. [42] have found that exercise program accelerates the circulation of body fluid to tissues and increases both skeletal muscle volume, which renders activities of daily living easier to perform and therefore less painful [43]. In addition to this, exercise may improve pain threshold via gradually increasing the range of motion and skeletal muscle strength in patients with musculoskeletal symptoms [44, 45]. As a nonpharmacological treatment method, exercise is low intensity, convenient, and easy to accept and maintain for a long time by patients [46].

Our study has several limitations. The meta-analysis only searched studies published in Chinese and English, which may have resulted in an incomplete literature review. Additionally, statistical heterogeneity of results exists when pooling data. Sensitivity analysis was applied to detect potential sources of heterogeneity, which may have attributed to the variability in intervention models, duration, outcome measurements, and results of the exacted time point. To achieve strong consensus regarding the effect of exercise and avoid the observed heterogeneity, further clinical trials should be conducted in a more uniform manner. Lastly, it was quite difficult to identify the best exercise program based on the data in this meta-analysis. Therefore, the results of this review need to be interpreted cautiously. Comparing the effect of different interventions deserves further research to support practitioners in clinical decision-making. This is in accordance with a study reported by Buffart et al. [47], who stated that more personalized exercise programs geared toward specific health outcomes are needed to ensure the efficacy and efficiency of intervention.

## Conclusion

Exercise is a safe and effective way to improve musculoskeletal symptoms and QOL in breast cancer survivors undergoing treatment with AIs. In the future, it is necessary to determine whether one type of exercise is better than another for maximum effect on AIMSS and quality of life in breast cancer patients. In addition, there is a need for more research to understand how to maintain the positive effect of exercise after the exercise intervention is completed.

## Abbreviations

AIs: aromatase inhibitors
AIMSS: aromatase inhibitor-associated musculoskeletal symptoms
QOL: quality of life
